# A comparison of the efficacy of transplantation of bone marrow-derived mesenchymal stem cells and unrestricted somatic stem cells on outcome after acute myocardial infarction

**DOI:** 10.1186/scrt127

**Published:** 2012-09-13

**Authors:** Aidan Flynn, Xizhe Chen, Enda O'Connell, Timothy O'Brien

**Affiliations:** 1 Regenerative Medicine Institute (REMEDI), National Centre for Biomedical Engineering Science, National University of Ireland, Galway, Ireland; 2 Cardiology Division, Massachusetts General Hospital, 55 Fruit Street, Boston, MA, 02114, USA; 3 National Centre for Biomedical Engineering Science, National University of Ireland, Galway, Ireland

**Keywords:** Cardiac failure, cardiac repair, guiding, mesenchymal stem cell, myocardial infarction, pre-conditioned, stem cell, umbilical cord, unrestricted somatic stem cell

## Abstract

**Introduction:**

A number of questions remain unanswered in the field of cell therapy for acute myocardial infarction, including what is the optimal cell type, and can therapeutic efficacy be enhanced by conditioning regimens. In this study, we sought to address these questions by directly comparing the effect of bone marrow-derived mesenchymal stem cells and unrestricted somatic stem cells delivered 24 hours post-myocardial infarction and by determining if the therapeutic efficacy of unrestricted somatic stem cells could be enhanced by exposing the cells to guiding factors before cell transplantation.

**Methods:**

Unrestricted somatic stem cells were guided by exposure to 50 ng/mL basic fibroblast growth factor, 20 ng/mL hepatocyte growth factor and 20 ng/mL bone morphogenetic protein-2 for 24 hours. Using a Sprague-Dawley rat model of acute myocardial infarction, we transplanted cells by intramyocardial injection 24 hours post-myocardial infarction. Cardiac function was serially measured using echocardiography, and histological analyses of infarct morphology, angiogenesis and apoptosis were obtained. Transcriptomic and proteomic changes were assessed using microarray and real-time quantitative PCR.

**Results:**

When assessed 28 days after the myocardial infarction, the delivery of mesenchymal stem cells 24 hours post-myocardial infarction did not improve ejection fraction (*P = *0.19), and did not prevent the decline in ejection fraction observed in the absence of cell therapy (*P = *0.17). The administration of unrestricted somatic stem cells also did not improve ejection fraction (*P = *0.11), but did prevent a further decline in ejection fraction (*P = *0.001). Delivery of guided unrestricted somatic stem cells significantly improved ejection fraction (*P = *0.03). Guided unrestricted somatic stem cells restored function to a greater extent than mesenchymal stem cells (*P = *0.03). The infarct area (*P = *0.2), apoptosis (*P = *0.07) and angiogenesis (*P = *0.09) did not differ between groups. Microarray analysis revealed that, following pre-implantation guiding, the gene groupings of mitosis, signalling and angiogenesis were highly overrepresented, mediators of apoptosis were overrepresented, and cardiomyocyte-associated genes were not differentially expressed.

**Conclusions:**

These results suggest that guided unrestricted somatic stem cells have a moderate capacity to repair cardiac damage and that they are more effective than mesenchymal stem cells in restoring cardiac function after a myocardial infarction. The mechanism of the benefit was not fully elucidated in this study, but these observations may be mediated by favorable dysregulation of angiogenic and apoptotic gene groupings.

## Introduction

Acute myocardial infarction (MI) results in cardiomyocyte death and scar formation. The resulting impaired cardiac function leads to cardiac failure and premature death. Stem cell therapy has the potential to limit the extent of cardiac damage by accelerating the normal healing process, improving vascularization, inhibiting apoptosis, and potentially regenerating cardiac muscle [1-4]. The mechanisms of effect by which stem cells improve cardiac function are increasingly being understood, and it is generally acknowledged that a combination of actions play a complementary role. The ability of transplanted cells to engraft and transdifferentiate has been shown by a number of investigators [5-7], but the extent of engraftment is low, and probably cannot account for the magnitude of effect, suggesting that alternative mechanisms play at least as important a role. One such complementary mechanism is the paracrine effect, in that mesenchymal stem cells (MSC) may mediate the functional improvement through secretion of soluble cytokines and growth factors [8]. In view of the probable significant contribution of the paracrine effect, a number of genetic and pharmacologic approaches have been employed to further advance the effectiveness of cell therapy. For example, bone marrow-derived MSC transfected with the anti-apoptotic gene *Akt *and delivered via an intra-coronary route resulted in a greater improvement in cardiac function 4 weeks post-MI than delivery of unmodified MSC [9]. Separately, the exposure of MSC to a 'cardiopoietic cocktail' was shown to enhance the reparative capacity of MSC by promoting their differentiation into a cardiac progenitor [10].

An additional method to optimize the effect of cell therapy is through the use of alternative cell populations. Bone marrow-derived MSC are the prototypical stem cell population, and although generally effective, genetic modification strategies in some studies have been required to demonstrate a beneficial effect [9]. Cells with a wider differentiation potential may have a greater capacity to repair cardiac damage than MSC. One such cell type is the umbilical cord blood-derived unrestricted somatic stem cell (USSC). This is considered to be a precursor of MSC, has a different surface phenotype, a wider differentiation profile, and has the advantage of non-invasive collection [11]. USSC have been shown to improve cardiac function in small and large animal models through a combination of autocrine and paracrine effects [12,13], and USSC have immunosuppressive properties that may confer protection from immune rejection [14]. These favorable features may provide additional therapeutic effects over MSC; however, a direct comparison of the effectiveness of USSC and MSC has not been performed, and the effect of pre-conditioning regimens on USSC has not yet been studied.

In this study, we aim to address these questions. Specifically, in an animal model of the intramyocardial delivery of stem cells after MI, we compare the efficacy of MSC with USSC; and we examine whether pre-transplantation guidance of USSC improves their therapeutic efficacy.

## Materials and methods

### Animals

Female Sprague-Dawley rats (Harlan Laboratories, Blackthorn, UK) weighing 200 g were used in this study. The experiments were approved by the Clinical Research Ethics Committee of National University of Ireland, Galway, and were in compliance with the *Guide for the Care and Use of Laboratory Animals *as published by the US National Institutes of Health (National Institutes of Health publication no. 85-23, revised 1996). The experiments were performed under a license granted by the Department of Health and Children, in compliance with the *Cruelty to Animals Act - Revised, 1876*. All procedures were performed by personnel certified by LAST-Ireland in animal welfare.

### Isolation and culture of cells

Bone marrow cells were isolated from the femoral and tibial compartments of female Sprague-Dawley rats (Harlan Laboratories) and plated at a density of 1.5 × 10^8 ^in T175 culture flasks. The mesenchymal cell population was isolated based on plastic adherence. Cells were cultured in α- Minimum Essential Medium and F12 medium (Sigma-Aldrich, St. Louis, MO, USA) with 10% fetal bovine serum (PAA Laboratories, GmbH, Pasching, Austria) and 1% antibiotic/antimycotic at 37ºC in 5% CO_2_. Passage four cells were used in this experiment. Prior to administration, cells were resuspended in 250 µL serum-free F12 media (Sigma).

### Unrestricted somatic stem cells culture

Passage four human USSC (Cell-Eng Tech, Coralville, IA, USA) were cultured in BulletKit (Sigma). Prior to administration, cells were resuspended in 250 µL serum-free F12 media (Sigma). For *in vitro *experimentation, USSC were plated at a density of 4 × 10^5 ^cells/cm^2^. After 24 hours, the media was changed, and fresh media was supplemented with 50 ng/mL basic fibroblast growth factor (bFGF), 20 ng/mL hepatocyte growth factor (HGF) and 20 ng/mL bone morphogenetic protein-2 (BMP2) (all from R&D Systems, Minneapolis, MN, USA). For *in vivo *experimentation, USSC were guided in an identical fashion and were detached with trypsin 0.025% after 24 hours. Expansion media was supplemented with 50 ng/mL bFGF, 20 ng/mL HGF and 20 ng/mL BMP2 24 hours prior to cell administration.

### Myocardial infarct model and cell administration

MI was performed as previously described. Briefly, Sprague-Dawley rats were anesthetized with isoflurane reduced to 5% with oxygen, intubated, and maintained on 2% isoflurane for the duration of surgery. Following a lateral thoracotomy, the left anterior descending coronary artery was identified and ligated 2 mm distal to the left auricle with a 7-0 polypropylene suture. MI was confirmed by blanching of the myocardium distal to the suture. Animals were randomly assigned to receive 4 × 10^6 ^MSC 24 hours post-MI, 4 × 10^6 ^USSC 24 hours post-MI or 4 × 10^6 ^guided USSC (cUSSC) 24 hours post-MI. Cells were delivered in five equal aliquots by an intramyocardial injection to the border zone of the infarct. Control animals received an equal volume of F12 without cells, or no intervention post-MI. We assigned our groups as MSC, USSC, cUSSC and Media and MI only.

### Echocardiographic analysis

An echocardiogram was performed at baseline, and at 48 hours post-MI in the case of animals not receiving cells, and 24 hours after cell administration in those randomized to cell therapy. A third echocardiogram was performed at sacrifice, 28 ±2 days after cell administration. A short-axis image of the left ventricle at the level of the papillary muscles was recorded with a 10 Hz electronic phased-array transducer and a VIVID5 Ultrasound System(General Electric, Fairfield, CT,USA). Ejection fraction (EF) was calculated as {(LVEDD)^3 ^- (LVESD)^3^}/(LVEDD)^3^, where LVEDD is the left ventricular end-diastolic dimension and LVESD is the left ventricular end-systolic dimension. EF was expressed as a percentage, and animals were included in the study if their EF decreased by >15% from its baseline. One animal was excluded due to its EF varying by more than three standard deviations from the mean.

### Assessment of infarct size

Myocardial fibrosis was detected by Masson's trichrome staining at 28 ±2 days post-MI. Briefly, hearts were harvested, and 15 to 18 slices of 5 µm thickness were prepared from midcavity to apex. A similar region of each heart was photographed (Olympus DP70, Olympus Corporation, Tokyo, Japan) with the aid of an inverted bright-field research microscope (Olympus IX71, Olympus Corporation) at resolutions of 1.25×, 4× and 10×, and images were recorded using the programs Image ProPlus and Analysis D.

### Angiogenesis assay and terminal deoxynucleotidyltransferase-mediated 2'-deoxyuridine 5'-triphosphate nick end labelling analysis

Capillary density was assessed by immunohistochemical staining with Von Willebrand factor antibody (1:800, Abcam, Cambridge, MA, USA). Angiogenesis was assessed by measurement of radial diffusion and length density. Using Image ProPlus software, eight images were obtained and a grid of a known area was placed over each image. Calculation of the number of intersections of vessels with a marker on the grid allowed calculation of the radial diffusion and length density.

Apoptotic cardiomyocytes in the border zone of the ischemic region were evaluated by terminal deoxynucleotidyltransferase-mediated 2'-deoxyuridine 5'-triphosphate nick end labelling (TUNEL) assay with an *in situ *cell death detection kit (Chemicon S7100 kit, Chemicon, Temecula, CA, USA). The percentage of TUNEL-positive cells in the border zone and infarct zone were compared to the TUNEL-positive cells in the normal myocardium.

### *In vitro *phenotypic and genotypic analysis of unrestricted somatic stem cells

#### Immunofluorescence

Immunostaining for cardiac troponin T (1:750, Abcam) and β-myosin heavy chain (1:100, Abcam) was performed. Slides were counterstained with 4,6-diamino-2-phenylindole-containing mounting medium (Sigma).

#### Microarray

RNA was isolated (RNeasy Mini Kit, Qiagen, Hilden, Germany) and concentrated (RNeasy MinElute Cleanup Kit, Qiagen) in accordance with the manufacturer's guidelines. The integrity of RNA was evaluated with an Agilent 2100 Bioanalyzer (Agilent Technologies, Santa Clara, CA, USA). Samples with an RNA Integrity Number >9.0 were labelled and hybridized. Genome-wide expression profiling was carried out using whole human genome 4 × 44 k oligo microarrays (Agilent). Linear amplification from 500 ng total RNA and spike-in-controls (Agilent) was performed using the Agilent Low RNA Input Linear Amplification Kit Plus, one colour. Amplified RNA was directly labelled by incorporation of Cy3-labelled cytidine triphosphate. Labelled RNA was purified with RNeasy Mini spin columns (Qiagen) and 1.65 μg labelled RNA was used for chemical fragmentation and hybridization (Gene Expression Hybridization Kit, Agilent). Assembly of the gasket/slide-sandwich in the hybridization chamber (Agilent), and hybridization in the Microarray Hybridization oven (Agilent) was performed according to manufacturer instructions. Slides were scanned using an Agilent DNA Microarray Scanner. Data extraction of the resulting array images was performed using the Feature Extraction software (Agilent, Version 9.1) and GeneSpring GX v11 (Agilent) was used for statistical analysis. All samples included in downstream analysis were required to pass Agilent's QC Metrics. The global gene list was filtered to remove non-expressed genes flagged as absent. To detect differentially expressed genes, a Welch T-test using Benjamini and Hochberg multiple testing correction with an adjusted *P*-value <0.05 was used, along with a fold change cut off of 2.0. Functional analysis was performed using the Database for Annotation, Visualisation and Integrated Discovery [15,16]. All microarray data are MIAME compliant, as detailed on the Microarray Gene Expression Data Society website [17]. The raw data have been deposited in the MIAME compliant database ArrayExpress [18] under accession number [E-MEXP-3638]. Data have been released in the public domain upon acceptance of this manuscript.

#### RNA profiling

Real-time quantitative PCR was performed using a TaqMan PCR kit (Applied BioSystems, Foster City, CA, USA) in triplicate. Threshold cycle values were determined using the 2-ΔΔCT method, normalized to human-specific β-actin (Applied BioSystems). Representative upregulated and downregulated genes, as identified on microarray analysis, and pre-defined cardiac-specific genes were analysed. These genes included kazal-type serine protease inhibitor domain-containing protein 1 (*KAZALD1 *[GenBank:NM_030929.4]; Hs00934805_m1), extracellular matrix protein 2, female organ and adipocyte specific (*ECM2*; [GenBank:NM_001197295]; Hs00946422_m1), v-erb-a erythroblastic leukemia viral oncogene homolog 4 (avian) (*ERBB4*; [GenBank:NM_001042599.1]; Hs00171783_m1), guanine nucleotide binding protein (*G protein*), alpha 14 (GNA14; [GenBank:NM_004297.3]; Hs00388871_m1), dehydrogenase/reductase (SDR family) member 2 (*DHRS2*; [GenBank:NM_005794.3]; Hs00195090_m1), galanin prepropeptide (*GAL*; [GenBank:NM_015973.3]; Hs01032384_m1), tetraspanin 2 (*TSPAN2*; [GenBank:NM_005725.4]; Hs00194836_m1), troponin T type 2 (cardiac) (*TNN2*; [GenBank:NM_000364.2]; Hs00945605_m1), GATA binding protein 4 (*GATA4*; [GenBank:NM_002052.3]; Hs00171403_m1), heart and neural crest derivatives expressed 1 (*HAND1*; [GenBank:NM_004821.2]; Hs00231848_m1), NK2 homeobox 5 (*NKX2.5*; [GenBank:NM_004387.3]; Hs00231763_m1) and myosin, heavy chain 7, cardiac muscle, beta (*MYH7*; [GenBank:NM_000257.2]; Hs01110632_m1).

### Statistical analysis

The echocardiographic data and infarct histology measurements were expressed as mean ±standard error (SE). Within group comparisons were analysed using one-sided t-test. Multiple groups were analysed with a one-sided analysis of variance and Bonferroni *post hoc *testing, using the statistical software PASW/SPSS® Version 18.0 (IBM, Chicago, IL, USA). A *P-*value of <0.05 was considered statistically significant. For analyses in which Levene's test of homogeneity of variances was significant (*P *<0.05), Brown-Forsythe and Welch testing was performed. Based on an expected improvement in EF of 10% (with a standard deviation of 9%) in the cUSSC group, 30 animals were required to provide a power of 90% and a two-sided alpha of 0.05 to detect this difference. Data analysis of the microarray study was performed as described above.

## Results

### Comparison of the effect of mesenchymal stem cells and unrestricted somatic stem cells administered 24 hours post-myocardial infarction

#### Assessment of cardiac function

**Figure 1:**
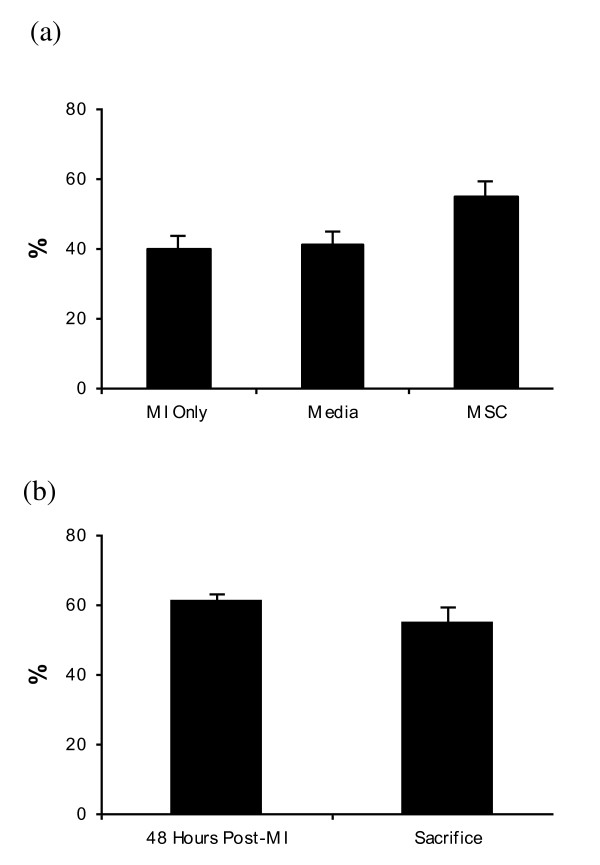
**Ejection fraction in mesenchymal stem cell group and control groups**. **(a) **The EF at sacrifice in the MSC group was not statistically significantly higher than the EF at sacrifice in the control groups. **(b) **There was a slight, but non-significant, decline in EF at sacrifice in the MSC group compared with the EF post-MI. MI Only: n = 5; Media: n = 6; MSC: n = 6. EF: ejection fraction; MI: myocardial infarction; MSC: mesenchymal stem cells.

The first measurement of cardiac function was performed 48 hours after MI. There was no difference between groups in the EF measured at this time-point, but the MSC group did have a trend towards a higher EF than the other groups. The administration of MSC post-MI did not have a significant effect on cardiac function, as measured by echocardiography. MSC administration resulted in an EF at sacrifice of 55.3% (SE: 3.9%; n = 6), which was not statistically significantly higher than the EF observed in the MI only (n = 5) group (39.7%; SE: 4.3%; *P = *0.17), or the Media (n = 6) group (41.2%; SE: 3.9%; *P = *0.24) at the same time-point (Figure 1a). The EF at sacrifice in the MSC group was 5.7% lower (SE: 5.2%; *P = *0.19) than the EF post-MI (Figure 1b).

**Figure 2:**
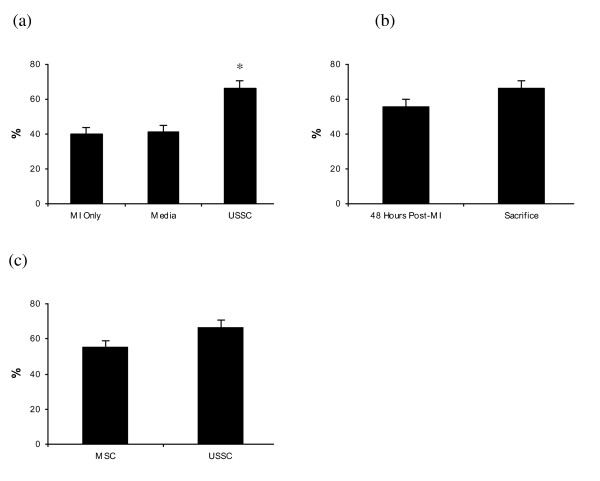
**Ejection fraction in unrestricted somatic stem cells group and control groups**. **(a) **The EF in the USSC group was significantly higher at sacrifice compared to the control groups (*P = *0.001). This is accounted for by the improvement in EF within this group (+11%), in comparison with the decline of approximately 5% in the control groups. **(b) **The improvement of 11% in the USSC group was not statistically significant (*P = *0.11). **(c) **There was no difference in the EF at sacrifice between the MSC group and the USSC group. MI Only: n = 5; Media: n = 6; MSC: n = 6; USSC: n = 6. EF: ejection fraction; MI: myocardial infarction; MSC: mesenchymal stem cells; USSC: unrestricted somatic stem cells.

The EF measured at sacrifice in the group receiving USSC (66.5%; SE: 3.9%; n = 6) was significantly greater than the EF measured at sacrifice in the control groups of MI only (*P = *0.001), and Media (*P = *0.001) (Figure 2a). The administration of USSC post-MI resulted in an improvement in EF of 11% (SE: 5.25%) at sacrifice, relative to the EF measured post-MI (Figure 2b), although this improvement was not statistically significant (*P = *0.11). The EF at sacrifice in the USSC group was 11.2% higher (SE: 5.5%) than the EF at sacrifice in the MSC group (55.3%; SE: 3.9%), but this difference was also not significant (*P = *0.76) (Figure 2c).

**Figure 3:**
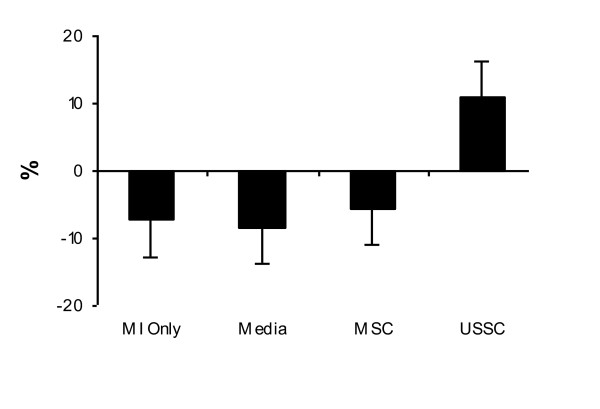
**Absolute change in ejection fraction across groups**. The USSC group was the only group to show an improvement in EF by the time of sacrifice. USSC delivery thus was considered to ameliorate the decline in EF observed in the control groups and the MSC group. MI Only: n = 5; Media: n = 6; MSC: n = 6; USSC: n = 6. EF: ejection fraction; MI: myocardial infarction; MSC: mesenchymal stem cells; USSC: unrestricted somatic stem cells.

As the EF post-MI in the MSC group showed a trend towards being higher than the EF in the other groups, we evaluated the change in EF within each group (the difference in EF at sacrifice relative to the EF post-MI), and compared this across groups. This 'absolute change' was not significantly different between the MSC group and the USSC group (*P = *0.48) (Figure 3). Thus, in this study, the administration of USSC ameliorated the decline in EF that is observed post-MI, and this effect was not observed following MSC delivery. However, USSC were not more effective than MSC in terms of restoring cardiac function.

#### Assessment of infarct size

**Figure 4:**
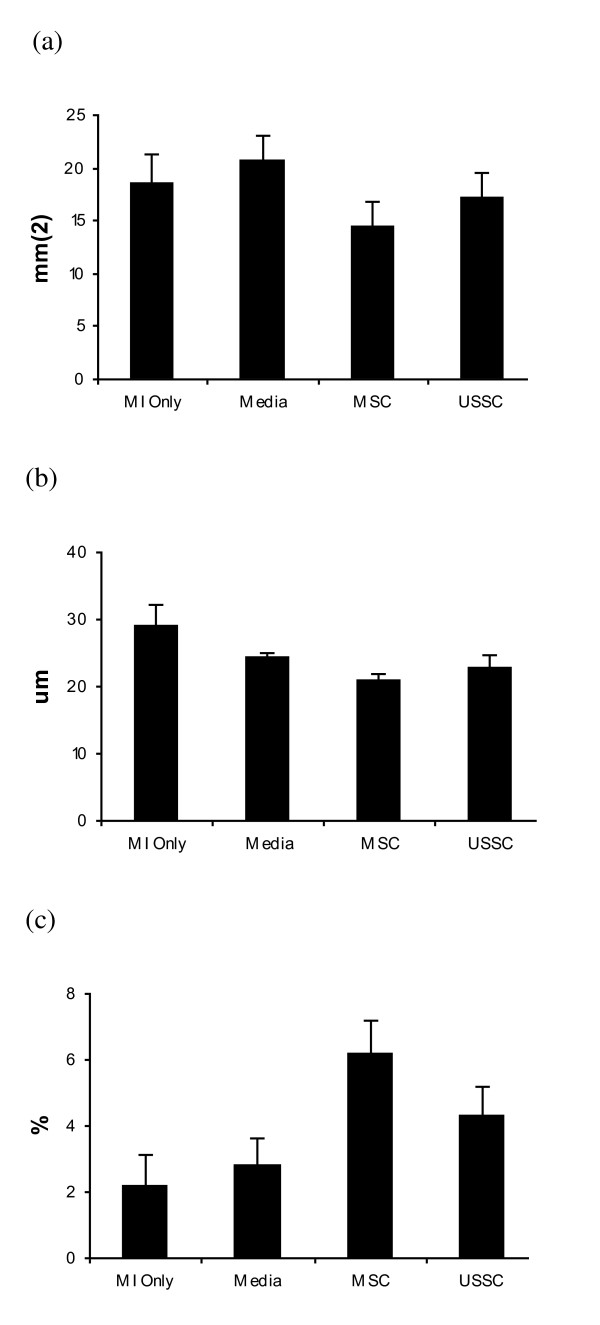
**Analysis of Infarct Size, Angiogenesis and Apoptosis In the Control Groups, MSC Group and USSC Group**. **(a) **Infarct size, **(b) **angiogenesis and **(c) **apoptosis. There was no significant difference in the infarct size, angiogenesis or apoptosis between groups. MI Only: n = 5; Media: n = 6; MSC: n = 6; USSC Group: n = 6. MI: myocardial infarction; MSC: mesenchymal stem cells; USSC: unrestricted somatic stem cells.

Infarct area was obtained by measuring infarct length and infarct width. The mean infarct areas of the MI only group (18.6 mm^2^; SE: 2.6 mm^2^) and the Media group (20.7 mm^2^; SE: 2.4 mm^2^) were not different from the infarct areas of the MSC group (14.4 mm^2^; SE: 2.4 mm^2^) or the USSC group (17.2 mm^2^; SE: 2.4 mm^2^). There was no difference in infarct area following administration of MSC or USSC (*P = *1.0) (Figure 4a).

#### Angiogenesis assay and TUNEL analysis

Angiogenesis was assessed by the extent of radial diffusion in the border zone of each infarct, and expressed as a mean. The average radial diffusion in the MI only group (29.0 μm; SE: 3.3 μm) and Media group (24.3 μm; SE: 0.7 μm) was compared with the MSC group (21.0 μm; SE: 0.9 μm) and the USSC group (22.9 μm; SE: 1.9 μm). Testing for non-parametric data revealed no significant difference between groups (Figure 4b).

In addition, the percentage of apoptotic cells in the border zone of infarcts, as assessed by TUNEL staining, was not different between groups (*P = *0.07), with similar percentages of TUNEL-positive cells in the MI only group (2.2%; SE: 0.9%), the Media group (2.8%; SE: 0.8%), MSC group (6.2%; SE: 1.0%) and USSC group (4.3%, SE: 0.9%) (Figure 4c).

### Effect of unrestricted somatic stem cells and guided unrestricted somatic stem cells administered at 24 hours post-myocardial infarction

#### Assessment of cardiac function

**Figure 5:**
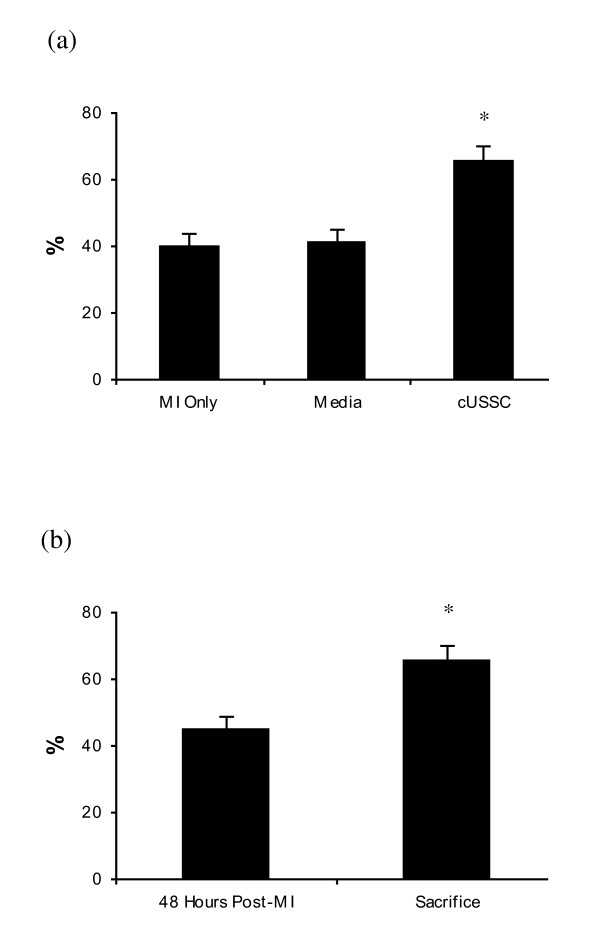
**Ejection fraction in the guided unrestricted somatic stem cells group and control groups**. **(a) **The EF at sacrifice in the cUSSC group was significantly higher than the EF at sacrifice in the control groups. **(b) **The EF at sacrifice in the cUSSC group was significantly higher than the EF at 48 hours post-MI. MI Only: n = 5; Media: n = 6; cUSSC Group: n = 5. cUSSC: guided unrestricted somatic stem cells; EF: ejection fraction; MI: myocardial infarction.

The administration of cUSSC resulted in a significantly higher EF at sacrifice (65.6%; SE: 4.3%; n = 5) relative to MI only and Media (*P = *0.003 for both comparisons) (Figure 5a). The administration of cUSSC resulted in an increase in EF of 20.7% (SE: 5.7%; *P = *0.03) at sacrifice, relative to the EF measured 24 hours after cell administration (Figure 5b). The absolute change in EF in the cUSSC group (an increase of 20.7%) was significantly greater than that observed in the control group of MI only (*P = *0.03) and Media (*P = *0.01).

**Figure 6:**
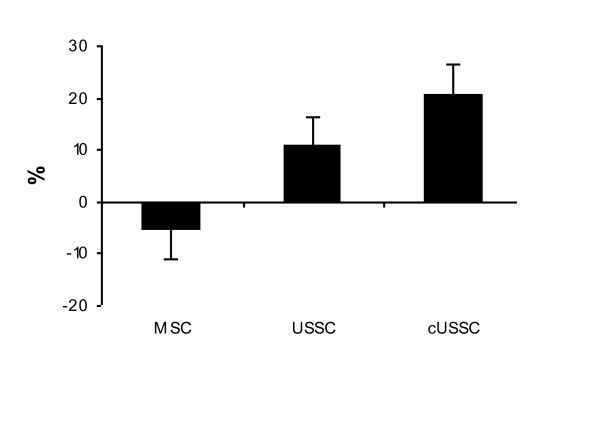
**Absolute change in ejection fraction across treatment groups**. The change in EF across the three treatment groups is presented. There is a slight decline in EF in the MSC group, and improvements in EF in the USSC and cUSSC groups. The extent of change in the cUSSC group (20.7%) was significantly greater than the change in the MSC group (-5.7%) (*P = *0.03). MSC: n = 6; USSC: n = 6; cUSSC: n = 5. cUSSC: guided unrestricted somatic stem cells; EF: ejection fraction; MSC: mesenchymal stem cells; USSC: unrestricted somatic stem cells.

Comparing the cUSSC group with both the unmodified USSC group and the MSC group, there was no difference in the absolute change between the cUSSC group and the unmodified USSC group (9.7%; SE: 7.8%, *P = *1.0), but there was a significant difference in the absolute change between the cUSSC group and the MSC group (*P = *0.03) (Figure 6). This demonstrates that, although USSC did not generate a significantly beneficial effect when compared to MSC, cUSSC induced a greater therapeutic effect, as measured by echocardiography.

#### Assessment of infarct size

**Figure 7:**
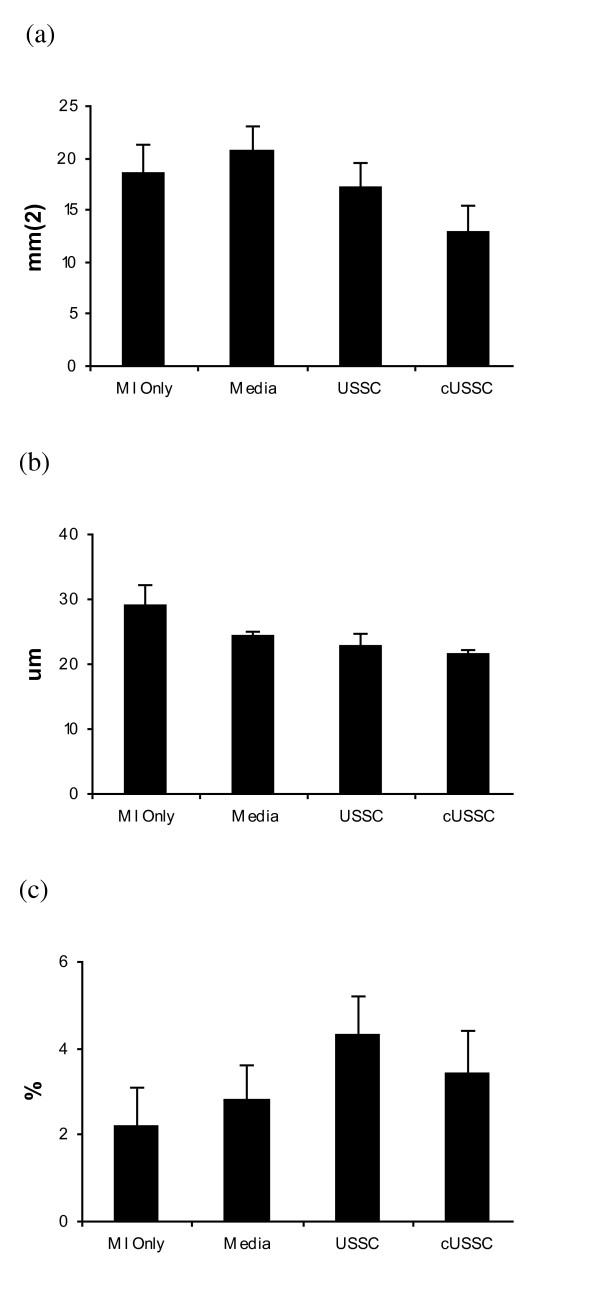
**Analysis of Infarct Size, Angiogenesis and Apoptosis In the Control Groups, USSC Group and cUSSC Group**. **(a) **Infarct size, **(b) **angiogenesis and **(c) **apoptosis. There was no significant difference in the infarct size, angiogenesis or apoptosis between groups. MI Only: n = 5; Media: n = 6; USSC: n = 6; cUSSC: n = 5. cUSSC: guided unrestricted somatic stem cells; MI: myocardial infarction; MSC: mesenchymal stem cells; USSC: unrestricted somatic stem cells.

**Figure 8:**
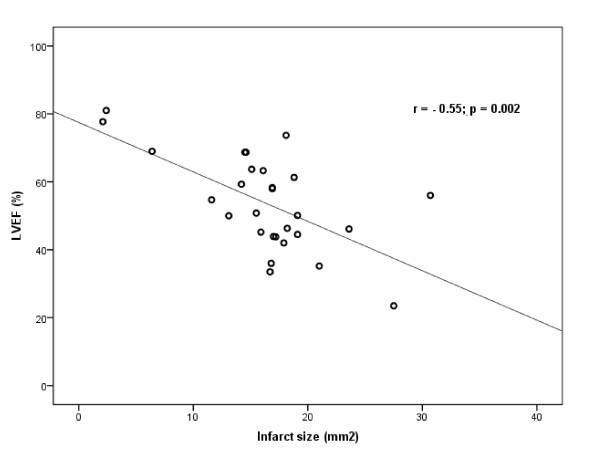
**Correlation between ejection fraction and infarct size**. Pooling all groups together, there is a significant correlation between EF and infarct size (r = -0.55; *P = *0.002). There was no evidence of clustering of groups. Of the two largest infarcts, one was observed in the USSC group, and one in the Media group. Of the three smallest infarcts, one each was observed in each of the cell groups. EF: ejection fraction; LVEF: left ventricular ejection fraction; USSC: unrestricted somatic stem cells.

The mean infarct area of the cUSSC group (12.8 mm^2^; SE: 2.6 mm^2^) was not significantly different from the infarct areas in the MI only group (*P = *1.0), the Media group (*P = *0.36), or the USSC group (*P = *1.0) (Figure 7a). Although there was no difference in infarct size between groups, this study was not powered *a priori *to detect this difference. Indeed, a comparison across all groups revealed a significant correlation between infarct area and EF (r = -0.55, *P = *0.002) (Figure 8), with no evidence of clustering. Of the two largest infarcts, one was observed in the USSC group, and one in the Media group. Of the three smallest infarcts, one each was observed in each of the cell groups.

#### Angiogenesis assay and TUNEL analysis

The radial diffusion in the group receiving cUSSC (21.6 μm; SE: 0.6 μm), was not significantly different than the radial diffusion in all other groups, as described above (Figure 7b).

The analysis of TUNEL staining also revealed the lack of effect of cUSSC on apoptosis (3.4%; SE: 1.0%) compared with all other groups (Figure 7c).

### *In vitro *phenotypic and genotypic analysis of unrestricted somatic stem cells

#### Immunofluorescence

The guiding regimen used in this study was 24 hour culture of USSC in media supplemented with 50 ng/mL bFGF, 20 ng/mL HGF and 20 ng/ml BMP2. This regimen did not result in enhanced expression of cardiac-specific markers (cardiac troponin T and β-myosin heavy chain) as assessed by immunofluorescence.

#### Microarray analysis

**Figure 9:**
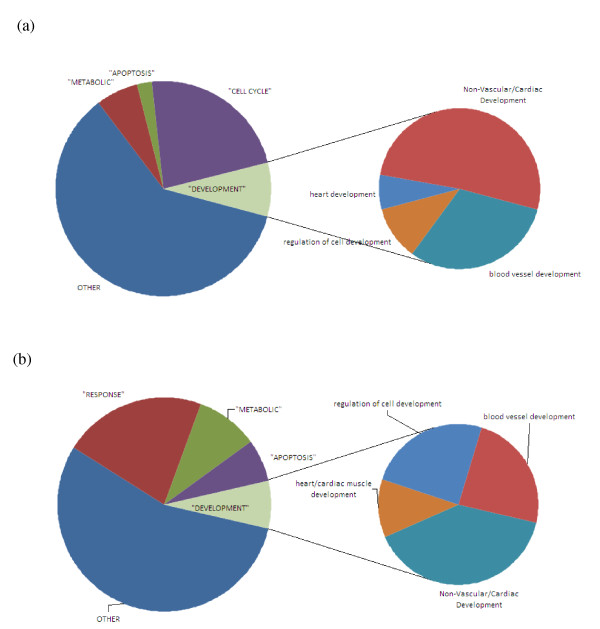
**Graphical representation of the (a) upregulated and (b) downregulated gene groupings based on enrichment scores**. The 388 upregulated and 348 downregulated genes are grouped according to their functional classification, and subdivided to specifically demonstrate the proportion involved in apoptosis and angiogenesis/cardiogenesis.

To determine genomic differences of cUSSC compared with unmodified USSC, their genome-wide gene expression profiles were studied using whole human genome 4 × 44 k oligo microarrays (Agilent). There was significant differential expression of 736 genes, with a fold change of ≥2.0 (adjusted *P*-value <0.05), with 388 upregulated and 348 downregulated. Functional analysis of differentially expressed genes was performed using the Database for Annotation, Visualisation and Integrated Discovery. From the functional annotation chart derived from this analysis, the three most significant terms are 'mitosis', 'cell division', and 'blood vessel development'. The functional terms 'vascular development' and 'blood vessel morphogenesis' were also highly represented, as were the terms 'angiogenesis' and 'regulation of angiogenesis'. Further evidence of the angiogenic profile of cUSSC was seen in the functional annotation chart, which revealed that the third most highly represented gene grouping was 'angiogenesis', after 'mitosis' and 'signalling'. Individual genes related to angiogenesis that were significantly upregulated included *fibroblast growth factor homologous factor 2 *(*FHF2*), which was upregulated approximately seven-fold; *fibroblast growth factor 13 *(*FGF13*), upregulated over four-fold; *placental growth factor *(*PGF*), coding for a vascular endothelial growth factor-like protein, upregulated over four-fold; and both angiopoietin-like 5 and platelet-derived growth factor subunit A-related peptide were upregulated over three-fold. Graphical presentations of these findings are presented in Figure 9.

Another of the most significantly expressed gene-sets included 'regulation of apoptosis' and 'programmed cell death'. Although in this study we did not observe a reduction in the extent of apoptosis after administration of cUSSC, the microarray finding suggests that cell death regulation may play a role in mediating the positive effects of cell therapy. Also of note is the observation that primitive and mature markers of cardiac differentiation, including *GATA4*, *Nkx2.5*, *β-myosin heavy chain *and *cardiac troponin T*, were not significantly differentially expressed in cUSSC. The absence of dysregulation of the aforementioned cardiac genes was confirmed by RT-PCR.

## Discussion

The evolution of cell therapy for cardiac disease has seen a gradual change in the cell populations that have been administered, from unfractionated bone marrow mononuclear cells in early reports [19] to bone marrow-derived MSC in subsequent reports [20], and recently to more cardiac-specific cell populations [10]. As the cell population has become more refined, the benefits of administration have been improving, with recent reports of the successful administration of cells of a cardiac specification (either autologous or guided), in both pre-clinical and clinical studies [21,22]. The variety of cell populations that have been studied demonstrates that the optimal cell population has not yet been defined, and also that cells that are more cardiac specific may be associated with a greater effectiveness. In this study, we observed results that are generally consistent with these prior observations. We studied three distinct cell populations: bone marrow-derived MSC, umbilical cord blood-derived USSC, and USSC that had been guided by pre-implantation exposure to factors considered to induce cardiac specification. The administration of cUSSC to an animal model of acute MI was associated with a beneficial effect on cardiac function.

Using echocardiography as a primary determinant of effectiveness, we observed that the delivery of MSC post-MI did not result in significant recovery of cardiac function post-MI, and it did not impact on the continued decline in cardiac function post-MI. Although many studies have shown that the delivery of bone marrow-derived MSC improves cardiac function, this observation has not been replicated by all investigators. For example, in one study, it was shown that hearts receiving unmodified MSC perform no better than untreated infarcted hearts [23]. The mechanism for this is postulated to be related to the intense inflammatory environment induced by infarction, in that transplanting cells into this environment may overwhelm the ability of these cells to survive. Modification of cells to enhance the expression of the pro-survival gene *Akt1 *was required before improvements in survival capacity were observed [9]. Furthermore, unmodified MSC, which do not have a cardiac specification, may have reduced ability to repair cardiac damage as they do not have a transcriptome that is conducive to cardiac repair. Pre-treating cells with a cardiopoietic cocktail has been shown to induce cardiac specification, and significantly enhances the cells' ability to restore cardiac function, as measured by echocardiography [10].

We evaluated the relative effectiveness of USSC, compared with MSC, to determine whether this cell population provides a greater potential for cardiac repair. We observed that the administration of USSC post-MI was only moderately effective, in that it prevents the decline in cardiac function that is observed in the absence of cell therapy. This echocardiographic outcome was not supported by significant changes in infarct size, angiogenesis or apoptosis however. Also, the administration of USSC was not more beneficial than the administration of MSC, and we cannot conclude that unmodified USSC are a substantially more effective therapy. Although there is evidence that umbilical cord blood-derived stem cells have intrinsic cardiomyogenic potential, which may provide a significant advantage over alternative cell populations [24], this potential advantage was not realized in our study.

In this study, we did not administer immunomodulatory agents to the animals receiving human USSC therapy, and therefore the possibility exists that an immune response could partially explain our results. However, while the administration of human MSC to animal models is associated with a significant immune response, umbilical cord blood-derived cells are more immune privileged and promote a much lower immune response [25]. Specifically, after the xenogeneic transplantation of umbilical- and bone marrow-derived stem cells, umbilical cord-derived stem cells were shown to be less immunogenic, cause less immune activation, and get rejected more slowly than bone marrow-derived cells. Separately, a recent review of the xenotransplantation of human adipose tissue-derived stem cells concluded that immunocompatability was observed in studies of the transplantation of human cells into rats, mice, dogs and rabbits [26]. Furthermore, in a study of the allogeneic activity of USSC [14], USSC are shown to be conditionally immunosuppressive and their immunological properties to be regulated by the local environment. Therefore, although in our study we did not administer immunomodulatory therapy, we do not believe that this had a significant impact on our observations.

Previous studies have demonstrated that cells that have been guided prior to implantation are more effective than unmodified cells. This finding has been observed in bone marrow-derived cells, and has not yet been described in USSC. The guiding factors used in this study (bFGF, HGF and BMP-2) were selected because, in embryonic tissue, they act on the primitive mesoderm to direct these cells down a cardiac pathway [27]. However, we did not induce dysregulation of genes involved in cardiac muscle generation. To successfully induce cardiomyogenesis, it is possible that either a wider selection of guiding factors is needed, or culture conditions need to be more cardio specific. It has previously been shown that treatment of MSC with similar factors (bFGF, BMP-2, insulin-like growth factor 1 resulted in an increase in expression of cardiac-specific markers only when co-cultured in the presence of neonatal cardiomyocytes [28]. Others have similarly reported that differentiation of bone marrow stromal cells into cells with a cardiac phenotype requires intercellular communication with myocytes [29], and that connexin-43, a protein required for gap junction formation, may mediate the cytoprotective effect of cell therapy. In our study, the absence of co-culture may have limited the ability of USSC to become cardiomyocytes.

In cUSSC, we observed transcriptomic changes that were consistent with upregulation of both pro-angiogenic and anti-apoptotic genes, suggesting that angiogenesis and programmed cell death play central roles in mediating the benefit of this therapy. Others have shown that new vessel formation is a central component of cardiac repair [30]; in the present study, functional annotation clustering revealed that angiogenic gene-sets were among the most upregulated clusters. Specific genes associated with angiogenesis that were upregulated in our study included *FHF2*, *FGF13 *and *PGF*, providing evidence of the enhanced angiogenic potential of the guided cells.

Consistent with the partial angiogenic genotype that was generated in cUSSC, we observed a modestly beneficial effect of cUSSC administration on cardiac function, as measured by echocardiography. Administration of this cell population improved cardiac function post-MI, and it also had a slightly greater effect than the administration of MSC. The mechanism for this benefit was not elucidated in the present study, as histological measurements of explanted hearts did not differ between groups. Although the transcription profile of cUSSC was consistent with them having a greater angiogenic potential, this observation was not supported by our histology. A possible explanation for this is that our study was powered on the basis of anticipated improvements in cardiac function as measured by echocardiography, rather than histological parameters. Indeed, while the difference in angiogenesis between groups was not statistically significant, with a *P-*value of 0.09, it is conceivable that a larger cohort may have allowed statistical significance to be achieved. Further research on this observation is warranted, and it will be necessary to include assessments of the efficiency of cell engraftment and cell survival. Although prior studies have shown engraftment and survival rates of 3% at 14 days after intramyocardial cell delivery [31], we did not directly assess this parameter in the present study. We also did not evaluate a group of animals receiving guided MSC, as this has been performed previously [27], and would not represent a novel aspect to our study. Given the greater plasticity of USSC [11], it could be assumed that the benefit of guiding USSC would exceed that observed following guided MSC delivery. Indeed, we observed that cardiac function improved following the administration of cUSSC, but as a direct comparative group was not included, we cannot conclude that guiding MSC would have had a lesser effect. With further study, an optimal guiding regime may be identified, which would allow a direct comparison across cell types.

The absence of an effect of cell therapy on infarct size has been described in a small number of studies, and is multifactorial [32]. Possible reasons for this lack of effect include insufficient cell survival in the peri-infarct milieu that allows for a decrease in infarct size being observed. While a small number of cells persist, and their presence is enough to affect cardiac function, they are not present in sufficient numbers to decrease the extent of fibrosis or induce the formation of new cardiac muscle. In this study, we did not evaluate the survival of USSC in cardiac tissue. Another possible reason for the absence of an observed benefit was the range of infarct sizes observed within each group. Our study was primarily powered to detect a change in EF using echocardiography, and was not powered to assess infarct size *a priori*. However, we did observe that there was a significant correlation between infarct size and EF that suggests that the inclusion of a larger number of animals may have allowed detection of significant decreases in infarct size in line with significant increases in EF. Finally, there is no consensus regarding the optimal dose of cells (and indeed route of administration [33]), and although we have previously investigated alternative doses and found the dose used in this study to be optimal, it remains possible that lower doses in small animals may be more beneficial.

## Conclusions

There is a moderately beneficial effect on cardiac function after the administration of cUSSC in an animal model of acute MI. This benefit is greater than that observed after the administration of MSC, using cardiac function measured by echocardiography as the marker of improvement. In this study, we did not demonstrate that infarct morphology differed between groups, but the cUSSC group did have significant favourable dysregulation of angiogenic and apoptotic gene-sets, which may have contributed to their effectiveness. Based on the results of this study, further research on the therapeutic effect of USSC and cUSSC is warranted.

## Abbreviations

bFGF: basic fibroblast growth factor; BMP2: bone morphogenetic protein 2; cUSSC: guided unrestricted somatic stem cells; EF: ejection fraction; HGF: hepatocyte growth factor; MI: myocardial infarction; MSC: mesenchymal stem cells; RT-PCR: reverse transcriptase polymerase chain reaction; SE: standard error; TUNEL: terminal deoxynucleotidyltransferase-mediated 2'-deoxyuridine 5'-triphosphate nick end labelling; USSC: unrestricted somatic stem cells.

## Competing interests

TOB has received education grants from Pfizer, Novartis and Merck Ltd, has received research grants from Medtronic, and is a founder, director and equity holder in Orbsen Therapeutics. The other authors declare that they have no competing interests.

## Authors' contributions

AF carried out all cell culture, performed and interpreted all echocardiographs, performed the microarray and PCR experiments, and drafted the manuscript. XC performed all surgical procedures and critically revised the manuscript. EOC participated in experimental design, microarray and PCR experiments and was involved in drafting the manuscript. TOB conceived of the study, participated in its design and coordination and critically revised the manuscript. All authors read and approved the final manuscript for publication.

## References

[B1] Gnecchi M Zhang Z Ni A Dzau VJ Paracrine mechanisms in adult stem cell signaling and therapy Circ Res 2008 103 1204 1219 10.1161/CIRCRESAHA.108.176826 19028920 PMC2667788

[B2] Kinnaird T Stabile E Burnett MS Shou M Lee CW Barr S Fuchs S Epstein SE Local delivery of marrow-derived stromal cells augments collateral perfusion through paracrine mechanisms Circulation 2004 109 1543 1549 Erratum in Circulation 2005, **112:**e73 10.1161/01.CIR.0000124062.31102.57 15023891

[B3] Behfar A Terzic A Optimizing adult mesenchymal stem cells for heart repair J Mol Cell Cardiol 2007 42 283 284 10.1016/j.yjmcc.2006.11.003 17174974

[B4] Meyer GP Wollert KC Lotz J Steffens J Lippolt P Fichtner S Hecker H Schaefer A Arseniev L Hertenstein B Ganser A Drexler H Intracoronary bone marrow cell transfer after myocardial infarction: eighteen months' follow-up data from the randomized, controlled BOOST (BOne marrOw transfer to enhance ST-elevation infarct regeneration) trial Circulation 2006 113 1287 1294 10.1161/CIRCULATIONAHA.105.575118 16520413

[B5] Toma C Pittenger MF Cahill KS Byrne BJ Kessler PD Human mesenchymal stem cells differentiate to a cardiomyocyte phenotype in the adult murine heart Circulation 2002 105 93 98 10.1161/hc0102.101442 11772882

[B6] Shake JG Gruber PJ Baumgartner WA Senechal G Meyers J Redmond JM Pittenger MF Martin BJ Mesenchymal stem cell implantation in a swine myocardial infarct model: engraftment and functional effects Ann Thorac Surg 2002 73 1919 1925 10.1016/S0003-4975(02)03517-8 12078791

[B7] Quevedo HC Hatzistergos KE Oskouei BN Feigenbaum GS Rodriguez JE Valdes D Pattany PM Zambrano JP Hu Q McNiece I Heldman AW Hare JM Allogenic mesenchymal stem cells restore cardiac function in chronic ischemic cardiomyopathy via trilineage differentiation capacity Proc Natl Acad Sci USA 2009 106 14022 14027 10.1073/pnas.0903201106 19666564 PMC2729013

[B8] Gharaibeh B Lavasani M Cummins JH Huard J Terminal differentiation is not a major determinant for the success of stem cell therapy - cross-talk between muscle-derived stem cells and host cells Stem Cell Res Ther 2011 2 31 10.1186/scrt72 21745421 PMC3219062

[B9] Gnecchi M He H Liang OD Melo LG Morello F Mu H Noiseux N Zhang L Pratt RE Ingwall JS Dzau VJ Paracrine action accounts for marked protection of ischemic heart by Akt-modified mesenchymal stem cells Nat Med 2005 11 367 368 10.1038/nm0405-367 15812508

[B10] Behfar A Yamada S Crespo-Diaz R Nesbitt JJ Rowe LA Perez-Terzic C Gaussin V Homsy C Bartunek J Terzic A Guided cardiopoiesis enhances therapeutic benefit of bone marrow human mesenchymal stem cells in chronic myocardial infarction J Am Coll Cardiol 2010 56 721 734 10.1016/j.jacc.2010.03.066 20723802 PMC2932958

[B11] Kögler G Sensken S Airey JA Trapp T Müschen M Feldhahn N Liedtke S Sorg RV Fischer J Rosenbaum C Greschat S Knipper A Bender J Degistirici O Gao J Caplan AI Colletti EJ Almeida-Porada G Müller HW Zanjani E Wernet P A new human somatic stem cell from placental cord blood with intrinsic pluripotent differentiation potential J Exp Med 2004 200 123 135 10.1084/jem.20040440 15263023 PMC2212008

[B12] Iwasaki H Kawamoto A Willwerth C Horii M Oyamada A Akimaru H Shibata T Hirai H Suehiro S Wnendt S Fodor WL Asahara T Therapeutic potential of unrestricted somatic stem cells isolated from placental cord blood for cardiac repair post myocardial infarction Arterioscler Thromb Vasc Biol 2009 29 1830 1835 10.1161/ATVBAHA.109.192203 19679830

[B13] Kim BO Tian H Prasongsukarn K Wu J Angoulvant D Wnendt S Muhs A Spitkovsky D Li RK Cell transplantation improves ventricular function after a myocardial infarction: a preclinical study of human unrestricted somatic stem cells in a porcine model Circulation 2005 112 I96 I104 10.1161/CIRCULATIONAHA.104.500256 16159872

[B14] Winter M Wang XN Daubner W Eyking A Rae M Dickinson AM Wernet P Kögler G Sorg RV Suppression of cellular immunity by cord blood-derived unrestricted somatic stem cells is cytokine-dependent J Cell Mol Med 2009 13 2465 2475 10.1111/j.1582-4934.2008.00566.x 19175687 PMC6512378

[B15] Huang da W Sherman BT Lempicki RA Systematic and integrative analysis of large gene lists using DAVID bioinformatics resources Nature Protocols 2009 4 44 10.1038/nprot.2008.211 19131956

[B16] Huang DW Sherman BT Lempicki RA Bioinformatics enrichment tools: paths toward the comprehensive functional analysis of large gene lists Nucleic Acids Res 2009 37 1 13 10.1093/nar/gkn923 19033363 PMC2615629

[B17] Microarray Gene Expression Data Society http://www.mged.org/Workgroups/MIAME/miame.html

[B18] ArrayExpress Archive http://www.ebi.ac.uk/microarray-as/ae/

[B19] Kocher AA Schuster MD Szabolcs MJ Takuma S Burkhoff D Wang J Homma S Edwards NM Itescu S Neovascularization of ischemic myocardium by human bone-marrow-derived angioblasts prevents cardiomyocyte apoptosis, reduces remodelling and improves cardiac function Nat Med 2001 7 430 436 10.1038/86498 11283669

[B20] Price MJ Chou CC Frantzen M Miyamoto T Kar S Lee S Shah PK Martin BJ Lill M Forrester JS Chen PS Makkar RR Intravenous mesenchymal stem cell therapy early after reperfused acute myocardial infarction improves left ventricular function and alters electrophysiologic properties Int J Cardiol 2006 111 231 239 10.1016/j.ijcard.2005.07.036 16246440

[B21] Bolli R Chugh AR D'Amario D Loughran JH Stoddard MF Ikram S Beache GM Wagner SG Leri A Hosoda T Sanada F Elmore JB Goichberg P Cappetta D Solankhi NK Fahsah I Rokosh DG Slaughter MS Kajstura J Anversa P Cardiac stem cells in patients with ischemic cardiomyopathy (SCIPIO): initial results of a randomised phase 1 trial Lancet 2011 378 1847 1857 10.1016/S0140-6736(11)61590-0 22088800 PMC3614010

[B22] Bartunek J Wijns W Dolatabadi D Vanderhayden M Dens J Ostojic M Behfar A Henry S Tendera M Waldman S C-Cure multicenter trial: lineage specified bone marrow derived cardiopoietic mesenchymal stem cells for treatment of ischemic cardiomyopathy J Am Coll Cardiol 2011 57 E200 10.1016/S0735-1097(11)60200-3

[B23] Gnecchi M He H Melo LG Noiseaux N Morello F de Boer RA Zhang L Pratt RE Dzau VJ Ingwall JS Early beneficial effects of bone marrow-derived mesenchymal stem cells overexpressing Akt on cardiac metabolism after myocardial infarction Stem Cells 2009 27 971 979 10.1002/stem.12 19353525 PMC2873075

[B24] Prat-Vidal C Roura S Farré J Gálvez C Llach A Molina CE Hove-Madsen L Garcia J Cinca J Bayes-Genis A Umbilical cord blood-derived stem cells spontaneously express cardiomyogenic traits Transplant Proc 2007 39 2434 2437 10.1016/j.transproceed.2007.06.016 17889212

[B25] Deuse T Stubbendorff M Tang-Quan K Phillips N Kay MA Eiermann T Phan TT Volk HD Reichenspurner H Robbins RC Schrepfer S Immunogenicity and immunomodulatory properties of umbilical cord lining mesenchymal stem cells Cell Transplant 2011 20 655 667 10.3727/096368910X536473 21054940

[B26] Lin CS Lin G Lue TF Allogeneic and xenogeneic transplantation of adipose-derived stem cells in immunocompetent recipients without immunosuppressants Stem Cells Dev 2012 in press 10.1089/scd.2012.0176 PMC3806387 22621212

[B27] Bartunek J Croissant JD Wijns W Gofflot S de Lavareille A Vanderheyden M Kaluzhny Y Mazouz N Willemsen P Penicka M Mathieu M Homsy C De Bruyne B McEntee K Lee IW Heyndrickx GR Pretreatment of adult bone marrow mesenchymal stem cells with cardiomyogenic growth factors and repair of the chronically infarcted myocardium Am J Physiol Heart Circ Physiol 2007 292 H1095 H1104 17056665 10.1152/ajpheart.01009.2005

[B28] Hahn JY Cho HJ Kang HJ Kim TS Kim MH Chung JH Bae JW Oh BH Park YB Kim HS Pre-treatment of mesenchymal stem cells with a combination of growth factors enhances gap junction formation, cytoprotective effect on cardiomyocytes, and therapeutic efficacy for myocardial infarction J Am Coll Cardiol 2008 51 933 943 10.1016/j.jacc.2007.11.040 18308163

[B29] Krysko DV Leybaert L Vandenabeele P D'Herde K Gap junctions and the propagation of cell survival and cell death signals Apoptosis 2005 10 459 469 10.1007/s10495-005-1875-2 15909108

[B30] Tang YL Zhao Q Zhang YC Cheng L Liu M Shi J Yang YZ Pan C Ge J Phillips MI Autologous mesenchymal stem cell transplantation induce VEGF and neovascularization in ischemic myocardium Regul Pept 2004 117 3 10 10.1016/j.regpep.2003.09.005 14687695

[B31] Freyman T Polin G Osman H Crary J Lu M Cheng L Palasis M Wilensky RL A quantitative, randomized study evaluating three methods of mesenchymal stem cell delivery following myocardial infarction Eur Heart J 2006 27 1114 1122 16510464 10.1093/eurheartj/ehi818

[B32] Holmes JW Borg TK Covell JW Structure and mechanics of healing myocardial infarcts Annu Rev Biomed Eng 2005 7 223 253 10.1146/annurev.bioeng.7.060804.100453 16004571

[B33] Bui QT Gertz ZM Wilensky RL Intracoronary delivery of bone-marrow-derived stem cells Stem Cell Res Ther 2010 1 29 10.1186/scrt29 20863415 PMC2983442

